# Phosphodiesterases Maintain Signaling Fidelity via Compartmentalization of Cyclic Nucleotides

**DOI:** 10.1152/physiol.00040.2013

**Published:** 2014-03

**Authors:** Oliver Lomas, Manuela Zaccolo

**Affiliations:** ^1^Department of Physiology Anatomy and Genetics, University of Oxford, Oxford, United Kingdom; and; ^2^Department of Cardiovascular Medicine, University of Oxford, Oxford, United Kingdom

## Abstract

Novel technological advances have improved our understanding of how cyclic nucleotides are able to convey signals faithfully between cellular compartments. Phosphodiesterases play a crucial role in shaping these signals in health and disease. The concept of compartmentalization is guiding the search for therapies that have the potential to offer greater efficacy and tolerability compared with current treatments.

In 1958, Sutherland and Rall (55) identified cyclic adenosine monophosphate (cAMP) as an inducer of protein phosphorylation in liver, skeletal muscle, heart, and brain tissue in response to hormones such as epinephrine and glucagon. From these observations, a paradigm developed of an extracellular “first messenger,” such as a hormone, which binds a receptor on the plasma membrane that in turn stimulates a cascade of intracellular signaling events via a “second messenger,” such as cAMP. From Sutherland and Rall's early observations, the biological significance of second messenger signaling grew rapidly as cAMP was found to mediate many signaling events in a diversity of tissues and even in prokaryotes (40). The discovery of cyclic guanosine monophosphate (cGMP) in the 1970s as a key second messenger in photo-transduction in the retina (44) broadened the concept to that of cyclic nucleotide signaling. Since these early observations, it has become apparent that cyclic nucleotide second messengers mediate numerous physiological functions, such as the force (inotropy) and frequency (chronotropy) of contraction of the heart (71), photo-transduction in the retina (44), glycogenolysis in the liver (47), and even control of DNA replication as part of mitotic cell division (75).

The phosphodiesterase (PDE) family of enzymes catalyze the hydrolytic degradation of cAMP and cGMP. PDEs are encoded by 21 genes from which are derived 11 different families, designated PDE 1–11 according to their structure, kinetics, substrate specificity, and regulatory mechanisms (31). As the sole means of terminating a cyclic nucleotide-dependent signal, PDEs provide a mechanism for controlling intracellular concentrations of cyclic nucleotides. This property has been exploited by a number of pharmacological agents for therapeutic effect. For example, aminophylline, which nonselectively inhibits PDEs to potentiate β-adrenoceptor signaling in airway smooth muscle, exerts clinically beneficial bronchodilatation in the context of chronic obstructive pulmonary disease (COPD) (64). However, the indiscriminate action of aminophylline on different PDE isoforms across different tissues contributes to its major adverse side effect of cardiac arrhythmia (52), which narrows its therapeutic window.

In an attempt to improve tolerability, drugs have been developed that target individual families of PDE. In particular, the PDE4 family of enzymes has been implicated in the pathogenesis of airway diseases such as COPD and asthma. PDE4 exclusively hydrolyses cAMP and is encoded by four separate genes that give rise to subfamilies A to D (11). In the case of COPD, the PDE4D isoenzyme has been found to be expressed by the leukocytes that drive the inflammatory component of the condition (6). Specific inhibitors of PDE4, such as roflumilast, have been developed for the maintenance treatment of COPD. However, PDE4 inhibitors, as a class of drug, are still hampered by their propensity to cause nausea and emesis (60).

Inhibition of PDE5 by sildenafil or tadalafil has proved clinically useful for augmenting vasodilatation for the treatment of erectile dysfunction. Similar effects of the PDE5 inhibitors on the pulmonary circulation have also been exploited for the treatment of pulmonary arterial hypertension (50). Despite the relative selectivity of the current generation of PDE5 inhibitors, unwanted effects are still encountered, such as a blue-tinged visual disturbance due to homology between PDE5 and the PDE6 isoform found in retinal photo-transduction (22).

The apparent lack of specificity of second messengers was a concern from early in the development of the field. Sutherland's initial observations of cAMP signaling being involved in multiple cellular functions encountered significant opposition from his contemporaries: How could such a seemingly ubiquitous signaling molecule mediate such a large variety of specific effects within cells? Indeed, in 1971, Rall (54) contemplated the “unsatisfying picture of the catalytic subunit of protein kinase swimming about, happily phosphorylating a variety of cellular constituents whether they need it or not.” From this, he presciently suggested the existence of “protein complexes” that “may become associated with other cellular components” to confer specificity of action. The aim of this review is to describe current understanding of how cyclic nucleotide signaling may be finely controlled and targeted, in particular through the localized action of PDEs.

## Compartmentalization of Cyclic Nucleotide Signaling

Compartments of the cell were originally described on the basis of visible membrane-delimited organelles such as the Golgi apparatus or mitochondria. However, compartmentalization may not only be considered to be structural but also as functional domains that are temporally and spatially regulated. Functional and biochemical evidence of segregated cyclic nucleotide signaling pathways was presented by Brunton et al. (14, 15), who described localized cAMP signaling in the heart. When isoproterenol was applied to cellular homogenates of rabbit heart, cAMP accumulation and PKA activation were recorded in both soluble and particulate subcellular fractions. However, prostaglandin E1 (PGE_1_) increased cAMP accumulation and PKA activity in the soluble fraction only. At the time of the experiments, cyclic AMP concentration ([cAMP]) was determined by an assay that takes advantage of the favorable binding of PKA to acidified cAMP (33), and PKA activity was determined using a histone phosphorylation technique (20). Both techniques made use of soluble and particulate fractions of cellular homogenates separated by centrifugation, which allowed the concept of compartmentalization to be proposed. To achieve coordinated cellular behavior, it became apparent that a sophisticated network, regulated both by subcellular location and time, was necessary for the cell to respond appropriately to the extracellular environment (63).

Proof of the existence of such functional compartments within cells and their dynamic regulation was limited by the techniques available, which only provided evidence of segregated cyclic nucleotide signaling pathways at a static, aggregated time point. The visualization of distinct functional compartments, rather than merely finding evidence of segregated pathways, was not possible until the development of live-cell imaging of cyclic nucleotide dynamics. These techniques provided improved spatial and temporal resolution, which allowed the formulation of new concepts in cyclic nucleotide signaling. The principle of live-cell imaging was first applied to intracellular calcium signaling with the application of sensitive indicators that revealed the presence of localized domains of calcium within cells (51). The first fluorescent biosensors for cAMP were developed in the early 1990s (1) but were superseded by genetically encoded biosensors, principally those that use the phenomenon of fluorescence resonance energy transfer (FRET) to measure dynamic, ratiometric changes in [cAMP] (73) or PKA activity (76) within cells.

## cAMP and cGMP as Second Messengers

The main effectors of cAMP and cGMP are protein kinases (PK) A and G, respectively. Other effectors include ion channels and, in the case of cAMP, the “exchange protein directly activated by cAMP” or EPAC (23). PKA comprises a heterotetramer of two catalytic (C) subunits bound to homo- or heterodimers of two regulatory (RI and RII) subunits (36, 66). PKA that contains RI subunits is referred to as PKA type I, and if the enzyme contains RII subunits, it is classified as PKA type II. Upon biding of cAMP to the R-subunit, each C subunit dissociates from the R-subunit dimer and becomes free to phosphorylate its targets. The in vivo pharmacokinetic properties of each R subunit differs in that RIα binds cAMP with much greater affinity than to RIIα or RIIβ (19, 26). Therefore, the consequences of a rise in [cAMP] may be encoded differently, according to which PKA isoform is involved. A sustained elevation in [cAMP] is likely to stimulate PKA-RII, whereas PKA-RI is likely to be stimulated by a transient, small elevation in [cAMP] (26, 68).

Cyclic GMP (cGMP) was first identified as a second messenger in photo-transduction in the retina (44). It is synthesized from guanosine triphosphate (GTP) by the action of the enzyme guanylyl cyclase (GC), which exists in soluble (sGC) or particulate (pGC) form, each of which is activated by different stimuli. The effectors of cGMP include protein kinase G (PKG), PDEs, which are also responsible for its degradation, and cyclic nucleotide gated (CNG) ion channels. PKG is a target of cGMP and bears structural similarity to PKA. However, rather than being a heterotetramer like PKA, PKG is a dimer comprising two identical subunits that remain associated upon binding cGMP (37).

The development of fluorescent reporters of specific components of nucleotide signaling, such as those that make use of the FRET phenomenon, have permitted the direct visualization of the limited diffusion of cyclic nucleotides that characterizes subcellular microdomains. Restricted domains of cAMP that are dependent on the activity of PDEs have been shown in the ventricular cardiomyocytes of neonatal rats in response to β-adrenergic receptor stimulation. Using a FRET biosensor, a striated pattern of [cAMP] consistent with the sarcomeric structure of cardiomyocytes was visualized, which was subsequently abolished upon inhibition of PDEs. This observation indicates an important role for these enzymes in limiting the spatial propagation of the signal (74). Using cAMP sensors that were targeted to the PKA-RI and PKA-RII isoforms, spatially restricted, independent compartments of cAMP have been visualized in rat ventricular cardiomyocytes, with distinct pools of cAMP activating different subsets of PKA and leading to the phosphorylation of different downstream targets (25). As with cAMP, evidence of nonuniformity of cGMP signaling has been derived from the use of FRET reporters (62) or CNG ion channels expressed in cardiomyocytes to monitor cGMP-gated membrane currents in response to manipulation of membrane-bound pGC compared with the cytosolic sGC (18, 62).

## Phosphodiesterases in Functional Compartments

**FIGURE 1.**
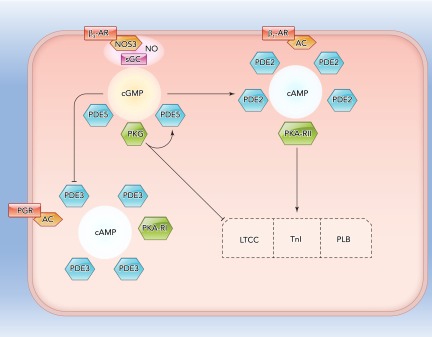
Cartoon depicting compartmentalization and cGMP- and cAMP-dependent signaling cross talk in a cardiomyocyte β-AR, β-adrenoceptor; LTCC, L-type calcium current; TnI, Troponin I; PLB, phospholamban; AC, adenylyl cyclase; sGC, soluble guanylyl cyclase; PGR, prostaglandin receptor. Arrows indicate stimulation. Blunt lines indicate inhibition.

Each PDE may be classified according to its substrate specificity (30). PDE4, 7, and 8 degrade cAMP alone; PDE5, 6, and 9 only degrade cGMP; and PDE1–3, 10, and 11 possess dual specificity since they degrade both cAMP and cGMP. A nonuniform distribution of cyclases (adenyly or guanylyl) and of PDEs allows gradients of cAMP or cGMP, respectively, to form within the cell such that cyclic nucleotide concentration is at its highest close to cyclase enzymes and at its lowest in the areas containing PDEs (FIGURE 1). The subcellular distribution of PDE3 and PDE4 isoforms was shown by Mongillo et al. (46), who also provided evidence of functional compartmentalization of PDE isoforms through their differential effects on β-adrenoceptor signaling. These observations contribute to the view of PDEs as not simply a means of terminating a cyclic nucleotide signal in a linear fashion but of defining a three-dimensional pool of cAMP in discrete subcellular domains.

The notion that the targeting of PDEs is important in cyclic nucleotide signaling is derived from work on the isoforms of PDE4. PDE4D3 has been found to be sequestered to particulate subcellular structures such as the sarcomeres of skeletal muscle via the large myomegalin protein (72). Serial truncation of multiple NH_2_-terminal portions of PDE4A5 has identified multiple regions of the protein that target the isoform to the cell membrane (8). In the HEK293 cell line, PDE4B and PDE4D isoforms are organized spatially and selectively modulate the concentration of cAMP in particular subcellular compartments (69), thereby creating discrete, localized pools of cAMP. PDE4B is localized in the plasma membrane, whereas PDE4D is mainly distributed in the cytosol. Through the use of the fluorescent live-cell imaging technique of FRET biosensors, PGE_1_ generated a larger cAMP response localized to the plasma membrane and nucleus than compared with the cytosol. The relative absence of cAMP from the cytosol is driven by activity of PDE4D because displacement of the endogenous PDE4D isoform disrupts this heterogeneity of [cAMP] (69).

An example of how recruitment of a PDE to a particular locale within the cell is vital to the efficient functioning of a signaling complex has been observed in cardiomyocytes. Upon binding of an agonist to the β_2_-adrenoceptor, there is activation of adenylyl cyclase and a concomitant increase in [cAMP], which in turn leads to stimulation of PKA. The β_2_-adrenoceptor is a target of PKA, and its phosphorylation switches the adrenoceptor from being coupled to a stimulatory G-protein (G_s_) to an inhibitory variant (G_i_), thus forming a feedback loop. However, Baillie et al. (5) have shown that β_2_-adrenoceptor also drives the recruitment of PDE4 via complex formation with β-arrestin, which was already known to block β-adrenoceptor G-protein-coupled activation. Through the recruitment of a PDE to a microdomain that includes the β_2_-adrencoceptor, the local cAMP concentration is expected to be lowered, thus counteracting the negative feedback inherent to cAMP-dependent PKA activation. Therefore, the intracellular concentration of cyclic nucleotides is strongly influenced not only by their synthesis via the relevant cyclases but also by the rate of degradation by PDEs.

## Subcellular Anchoring of the Components of Cyclic Nucleotide Signaling

Much of our understanding of compartmentalized cyclic nucleotide signaling is derived from the cAMP-PKA pathway. Type I PKA (i.e., containing RI subunits) is found mainly within the cytoplasm, whereas the majority of type II PKA (i.e., containing RII subunits) is associated with specific membranous cellular structures (67). The localization of these different isoforms approximates to the segregated PKA-dependent pathways observed by Brunton et al. (13) in the early 1980s. In addition to the different pharmacological characteristics of these isoforms, a particular focus has been placed on the localization of proteins that restrict them to particular intracellular domains and thereby increase the likelihood of interaction by the molecular components of the pathway. A-kinase anchoring proteins (AKAPs) are structurally unrelated, multivalent scaffolding proteins that have been classified as a family of >50 proteins based on their ability to bind PKA via an amphipathic helix (35, 43, 59). Regulatory proteins such as phosphatases and PDEs frequently form part of such spatially confined signaling complexes. AKAPs are classified as RI selective, RII selective, or dual specificity, depending on the R-subunit isoforms for which they show higher affinity of binding. The physical constraint of PKA isoforms in defined subcellular compartments allows for the activation of particular subsets of PKA and hence the phosphorylation of selected targets in response to specific stimuli that increase the intracellular concentration of cAMP.

The proximity of PKA to a specific substrate is vital to the effective functioning of the signaling complex. In rat ventricular myocytes, knockdown of AKAP18δ, an isoform that anchors PKA in proximity to its substrate phospholamban (PLB) at the sarcoplasmic reticulum (SR), was shown to affect the ability of the sarco-/endoplasmic reticulum Ca^2+^-dependent ATPase (SERCA) pump to replenish the SR with Ca^2+^ during myocyte relaxation (38).

**FIGURE 2.**
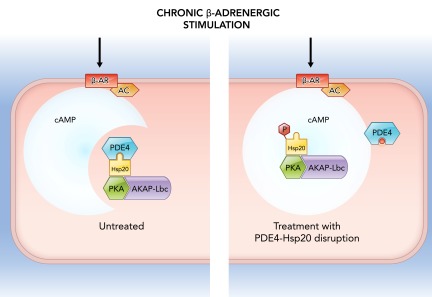
Cartoon depicting how interrupted subcellular PKA-cAMP microdomain signaling may affect cardiac hypertrophic growth Hsp20, heat shock protein 20. Red bar indicates disruptive peptide.

In the heart, the small heat-shock protein Hsp20 has been associated with protection against pathological hypertrophy, apoptosis, and ischemia-reperfusion injury (77), the pathological process that occurs when the blood supply is restored to previously ischemic tissue (34). A significant component of these pathological processes is the increased activity of PKA-dependent β-adrenoceptor-mediated signaling (29). The protective effects to the heart of Hsp20 only appear to manifest following phosphorylation at a PKA/PKG-dependent motif (28). PDE4 has been found to form a complex with Hsp20, which prevents Hsp20 from being phosphorylated under the resting conditions (58). Disruption of the interaction between Hsp20 and PDE4 by means of an exogenous peptide quashes PDE-dependent attenuation of phosphorylation of Hsp20 by PKA. This intervention had the anticipated result of diminishing the hypertrophic response of neonatal cardiomyocytes to chronic β-adrenergic stimulation (58). Hsp20 has also been shown to be associated with the anchoring protein AKAP-Lbc (27), which has already been implicated in cardiac hypertrophy (17). These observations suggest how specific targeting of signaling complexes may represent a novel opportunity for the treatment of heart failure (27) (FIGURE 2).

For many years, it has been known that cyclic nucleotides play an important role in the control of the cell cycle (75). Signaling via the cAMP/PKA pathway has been shown to be involved in many parts of the cell cycle (42). Evidence for different compartments of cAMP/PKA signaling being responsible for different cell cycle events has been shown by Terrin et al. (70). A global increase in cAMP is associated with an accumulation of cells in Gap 1 (G_1_) phase before DNA synthesis, whereas displacement of PDE4D3 from the centrosome, causing a local increase in [cAMP], leads to cell-cycle arrest at prophase of mitosis. Another example of the role played by localized PDEs has been described in a cancer cell model. The D5 isoform of PDE4 has been shown to interact with focal adhesion kinase (FAK) and the receptor for activated C-kinase (RAK). Displacement of the PDE isoform from the PDE4D5/RAK/FAK complex leads to a significantly less invasive phenotype (56).

As is found with cAMP signaling, localized cGMP signaling is achieved through the compartmentalization of its producer (guanylyl cyclase) and its downstream effectors PDEs and PKG. The role of cGMP as a second messenger was first identified in the highly organized signal transduction pathway of vertebrate photo-transduction in the retina that is dependent on cGMP acting directly on ion channels. Absorption of a photon of light by the photoreceptor rhodopsin in turn activates the G-protein transducin that subsequently stimulates PDE6 to catabolize cGMP to GMP and leads to closure of CNG channels responsible for the depolarizing “dark” current (7). PDE6, as found in rod photoreceptors, comprises a heterodimer of α- and β-catalytic subunits, which are each bound to an inhibitory γ-subunit. Prenylation of catalytic subunits binds the protein to the plasma membrane. However, the presence of a prenyl-binding protein renders PDE6 soluble, i.e., dissociating PDE6 from the membrane and thus preventing its activation by transducin and thereby desensitizing the response to light (48). The spatial proximity of these signaling proteins is vital to their function. Inhibition of PDE6 by the nominally selective PDE5 inhibitor tadalafil, causing visual color disturbance, highlights the necessity of specific PDE-mediated control of signal transduction (16).

The intracellular distribution and dynamics of cGMP signaling are less well understood compared with cAMP signaling, but insight has been gained in the myocardium and the vasculature, where nitric oxide (NO), derived from the nitric oxide synthase 3 (NOS3) isoform, is a potent stimulator of soluble guanylyl cyclase (sGC). In the failing myocardium, PDE5 expression is elevated compared with the normal heart (53). Immunocytochemistry in isolated cardiac myocytes has suggested that PDE5 is localized to the Z-bands, which represent the borders of the sarcomere and to which actin is bound. This localization is dependent on the presence of an intact eNOS-sGC signaling complex as the co-localization is abolished in the presence of chronic pharmacological inhibition of NOS3 or in NOS3^−/−^ cells (65). If cGMP binds a regulatory GAF domain of PDE5, a conformational change is induced, which increases the catalytic activity of PDE5 (78). This binding of cGMP to the GAF domain is augmented by PKG-dependent phosphorylation (21). Therefore, as the concentration of cGMP increases, PKG activity is stimulated, an action of which is to promote the catalysis of cGMP via PDE5, thus representing a negative feedback loop within a signaling complex (FIGURE 1). Such a localized signaling complex has been described, whereby sGC-derived cGMP is degraded through the action of PDE5, but cGMP generated by pGC is accumulated (18). These data provide evidence of segregated cGMP-dependent signaling pathways, which contribute to our understanding of the distinct effects of natriuretic peptides and NO donors on the heart.

## Cross Talk Between cAMP and cGMP Pathway

In addition to anchoring PKA isoenzymes, AKAPs aid the assembly of multi-protein signaling complexes that include other proteins such as PDEs, protein kinases, and protein phosphatases (43). In this way, AKAPs and PDEs facilitate signal transduction, signal termination, and cross talk with other signaling pathways and so organize cAMP signaling in both its temporal and spatial dimensions. PDEs are downstream targets for cGMP signals and provide a point at which cGMP-dependent signaling can influence cAMP-dependent signaling, a mechanism known as cross talk.

In isolated cardiomyocytes, β_1_-adrenoceptor stimulation results in greater cAMP synthesis in the PKA-RII compartment than the RI compartment, which leads to phosphorylation of modulators of cardiac contractility such as the L-type calcium channel (LTCC), PLB, and troponin I (TnI) (25). However, in the presence of an increase in intracellular sGC-derived cGMP, for example via β_3-_adrenoceptor activation, the generation of cAMP in the RI and RII compartments is inverted, such that [cAMP]_RI_ is augmented and [cAMP]_RII_ is attenuated (62). Stimulation of pGC by atrial natriuretic peptide (ANP) appears to diminish [cAMP]_RII_ selectively. These observations are accounted for by the different effects of cGMP on PDE2 and PDE3 isoforms in cardiomyocytes. When PDE2 binds cGMP, allosteric modification of the enzyme takes place, which increases the affinity of the enzyme for cAMP and thus improves the catalytic efficiency of cAMP degradation. This allosteric stimulation of cAMP hydrolysis by cGMP is more important, quantitatively, than its competitive inhibition of cAMP hydrolysis, although cAMP hydrolysis can be inhibited in vitro by cGMP at high (>50 μM) concentrations (9). In opposition to the effects of PDE2, the cAMP hydrolytic activity of PDE3 is inhibited upon binding of [cGMP] (49). Through such regulatory mechanisms and depending on the PDE involved, stimuli that elevate [cGMP] attenuate or enhance the cAMP signal.

This signaling cross talk provides a mechanism to explain how β_3_-adrenceptors exert a functionally antagonistic effect to β_1/2_-adrenoceptor signaling (24) in that they exert a mildly negatively inotropic effect, which is protective in pathological, excessive states of β_1/2_-adrenoceptor stimulation as found in heart failure. A further, distinct example of the inhibitory effect of PDE2 on cAMP-dependent signaling has been highlighted by the observation that mice that lack plasma membrane calcium ATPase 4 (PMCA4), which localizes nNOS to the sarcolemma, exhibit displacement of nNOS activity to the cytosol. This fails to drive PDE2-mediated cAMP hydrolysis, resulting in an elevation in [cAMP] (45). Another example of how disrupted localization of PDE3-dependent microdomains may contribute to pathological conditions is the observation that, when the phosphoinositide 3-kinase γ (PI3Kγ) regulatory component is rendered absent from cardiac myocytes by gene deletion, loss of the usual compartmentalization of PI3Kγ with β-adrenoceptors and PDE3 is observed (32). The subsequent increased PKA activity causes greater PLB and LTCC phosphorylation, with a concomitant rise in the likelihood of arrhythmia from a greater cytosolic calcium ion concentration (10).

Cross talk between the cAMP and cGMP signaling pathways through PDEs 2 and 3 has important physiological functions in tissues other than the heart. Platelets are enucleate, granulocytic fragments of megakaryocytes that are important mediators of thrombus formation and wound repair. NO is an important modulator of platelet function in thrombogenesis through actions on adhesion and recruitment at the site of injury (2), with the net effect being to inhibit thrombogenesis (4). NO, for example derived from the NOS3 isoform in the vasculature, drives the synthesis of cGMP via sGC to exert PKG-dependent effects on cellular function (41). Prostaglandins such as PGE_1_ and PGI_2_ are important mediators of platelet function that stimulate cAMP production (3). The potential for cross talk between cGMP and cAMP signaling pathways has been appreciated for some time, and it has become apparent that, as found in cardiomyocytes, cGMP modulation of PDE2 and 3 plays an important role in negative feedback control of cAMP concentration (39).

## Conclusions

Compartmentalization, the structural and functional restriction of signaling domains, allows distinct pools of cyclic nucleotide to interact with particular effectors. PDEs are key modulators of cyclic nucleotide signaling and hence represent attractive therapeutic targets for cardiovascular, respiratory (61), neurodegenerative (12), and inflammatory diseases (57). The corollary to this is that inhibitors that are applied without respect to functional localization elicit numerous unwanted effects, as is the case even with the current generation of more specific PDE inhibitors. As more is learned about the structural organization of individual signaling domains, the use of competing peptides or small molecules to displace a specific PDE isoform from a particular complex is an attractive means by which to test whether selective manipulation of pools of cyclic nucleotides, at specific locations, may avoid global off-target effects in other compartments and improve efficacy (58). Due to their ubiquity within signaling systems across tissues, PDEs present opportunities and constraints in the design of therapeutic agents, a challenge that may be met through the specific targeting of PDEs found within signaling cascades that have been implicated in pathology.

From the early discoveries relating to cyclic nucleotide signaling over 60 years ago, significant advances in our understanding of cyclic nucleotide signaling have been achieved. With our increasing understanding of the localized nature of cyclic nucleotide signaling, specific targeted therapy to subcellular domains represents not only a challenge but an opportunity to improve both therapeutic tolerability and efficacy.
